# Structural correlates for lexical efficiency and number of languages in non-native speakers of English

**DOI:** 10.1016/j.neuropsychologia.2012.02.019

**Published:** 2012-06

**Authors:** A. Grogan, ‘Ō. Parker Jones, N. Ali, J. Crinion, S. Orabona, M.L. Mechias, S. Ramsden, D.W. Green, C.J. Price

**Affiliations:** a Wellcome Trust Centre for Neuroimaging, Institute of Neurology, UCL, UK; b Anglia Ruskin University, Cambridge, UK; c Institute of Cognitive Neuroscience, UCL, UK; d Cognitive, Perceptual and Brain Sciences, UCL, UK

**Keywords:** Language, Efficiency, Lexical, MRI, Bilingual

## Abstract

We used structural magnetic resonance imaging (MRI) and voxel based morphometry (VBM) to investigate whether the efficiency of word processing in the non-native language (lexical efficiency) and the number of non-native languages spoken (2+ versus 1) were related to local differences in the brain structure of bilingual and multilingual speakers. We dissociate two different correlates for non-native language processing. Firstly, multilinguals who spoke 2 or more non-native languages had higher grey matter density in the right posterior supramarginal gyrus compared to bilinguals who only spoke one non-native language. This is interpreted in relation to previous studies that have shown that grey matter density in this region is related to the number of words learnt in bilinguals relative to monolinguals and in monolingual adolescents with high versus low vocabulary. Our second result was that, in bilinguals, grey matter density in the left pars opercularis was positively related to lexical efficiency in second language use, as measured by the speed and accuracy of lexical decisions and the number of words produced in a timed verbal fluency task. Grey matter in the same region was also negatively related to the age at which the second language was acquired. This is interpreted in terms of previous findings that associated the left pars opercularis with phonetic expertise in the native language.

## Highlights

► We dissociate structural correlates for two different non-native language skills. ► Number of languages spoken was associated with the posterior supramarginal gyrus. ► The efficiency of word use was associated with the left pars opercularis.

## 1 Introduction

Vocabulary is key to effective language use and structural neuroimaging studies have identified brain regions where grey matter density correlates with variations in vocabulary knowledge in both monolingual (Lee, Devlin, Shakeshaft, & Stewart, 2007; Richardson, Thomas, Filippi, Harth, & Price, 2010) and bilingual (Mechelli et al., 2004) speakers. In addition to vocabulary knowledge, everyday communication requires the timely retrieval and processing of words (i.e., lexical efficiency). Word production and word comprehension both require efficient selection and discrimination of the intended words from potentially competing words. Such processes typically implicate activation in left frontal lobe regions (Noppeney, Phillips, & Price, 2004; Thompson-Schill, D’Esposito, & Dan, 1999; Wagner, Pare-Blagoev, Clark, & Poldrack, 2001). If so, inter-subject variability in lexical efficiency may correlate with structural differences in left frontal regions that are distinct from the parietal (Lee et al., 2007; Mechelli et al., 2004) and temporal (Richardson et al., 2010) regions associated with vocabulary knowledge. We explore this possibility by conducting a structural magnetic resonance imaging (MRI) study of multilingual participants who varied in their lexical efficiency and the number of languages spoken.

Our study builds on the earlier work of Mechelli et al. (2004) who examined inter-subject variability in the brain structure of bilinguals relative to monolinguals, and also in a sample of Italian-English speakers who varied in their English vocabulary knowledge and the age at which they acquired English. Grey matter density in the posterior supramarginal gyrus (pSMG) was found to be higher for bilinguals than monolinguals and to covary positively with vocabulary knowledge and negatively with age of acquisition. These effects were stronger in left relative to right pSMG. A positive correlation between vocabulary knowledge and grey matter density in left and right pSMG was also observed in monolingual adolescents (Lee et al., 2007; Richardson et al., 2010) indicating that it is not unique to bilinguals. However, interestingly, this correlation was not found to be significant in monolingual adults (Richardson et al., 2010). Instead vocabulary knowledge in monolingual adults correlated with grey matter density in two left temporal regions (the left posterior superior temporal sulcus and left posterior temporo-parietal cortex) associated with written and auditory sentence processing.

Richardson et al. (2010) suggest that the effect of age on vocabulary knowledge may reflect the differential use of two distinct learning strategies: one formal and one contextual. Adolescents, and learners of a second language (as in Mechelli et al., 2004), may use an explicit (formal) learning strategy to increase their vocabulary knowledge, with this strategy involving the pSMG – an area that plausibly links sound and meaning (see Lee et al., 2007). In contrast, adult speakers may primarily use written or spoken sentence context to expand their vocabulary and so vocabulary knowledge may correlate with temporal lobe regions associated with sentence processing. Such a view is consistent with the idea that establishing the meaning of words involves experiential data like word referents and distributions over sentence contexts (Andrews, Vigliocco, & Vinson, 2009).

These prior studies provide good evidence that brain structure correlates with vocabulary knowledge, but do not indicate if these regions are sensitive to the efficiency of word use. Bilingual and multilingual speakers vary in the efficiency with which they use words and also in the age at which they acquired their second language. Both factors may influence or be influenced by different brain regions. Even the sheer number of languages learned might influence inter-subject variability in brain structure. To the best of our knowledge ours is the first study to compare the structural correlates of lexical efficiency and number of languages learnt. We first describe our measures and then our predictions and expectations.

We used two indices to derive a measure of lexical efficiency in English that was a non-native language for all our participants. First, as an index of the efficiency with which individuals produce single English words we used the letter fluency task. In this task, individuals generate as many words beginning with a certain letter as they can in one minute. The index was simply the number of correct words produced (as the base time was constant at one minute). Second, as an index of the efficiency with which individuals recognise written English words, participants performed a timed lexical decision task (with words derived from the Psycholinguistics Assessments of Language Processing in Aphasia, PALPA: Kay, Lesser, & Coltheart, 1992). Parallel to the fluency index, the lexical decision index was the percentage of correct lexical decisions divided by the mean correct response time. We created a single lexical efficiency score for each participant by identifying the principal component of the lexical decision and fluency indices using factor analysis (see Mechelli et al., 2004 for previous use of this method). For each participant we also recorded the number of languages they spoke and the age at which they acquired English, as these factors might also affect variations in regional grey matter density.

Timely retrieval and encoding of words requires discriminating and selecting the intended word from competing alternatives. Such requirements suggest that left frontal regions will play an important role (Noppeney et al., 2004; Thompson-Schill et al., 1999; Wagner et al., 2001). Functional neuroimaging studies also consistently report left inferior frontal activation during both lexical decision (Binder et al., 2003; Carreiras, Mechelli, & Price, 2006; Newman & Joanisse, 2011) and fluency tasks (Birn et al., 2010; Frith, Friston, Liddle, & Frackowiak, 1991; Meinzer et al., 2009; Mummery, Patterson, Hodges, & Wise, 1996; Phelps, Hyder, Blamire, & Shulman, 1997; Whitney, Grossman, & Kircher, 2009). A recent longitudinal study of second language learning (Stein et al., 2012) has also reported structural changes in the left inferior frontal gyrus that correlate with the increase in second language proficiency. Therefore we expected that our measure of English lexical efficiency might correlate with brain structure in the left inferior frontal cortex.

If bilinguals also rely on pSMG to produce timed responses then we may also find that pSMG grey matter correlates with lexical efficiency. If, on the other hand, this region reflects verbal knowledge learnt by linking phonological and semantic associations using explicit/formal strategies (Richardson et al., 2010) then it may not be sensitive to lexical efficiency. However, it should still be sensitive to the number of languages spoken (i.e., a difference between multilingual and bilingual speakers) because we would expect multilinguals to have learnt more vocabulary overall than bilinguals. Our participants differed in their native languages and so we also tested whether our results were influenced by the native language (European or non-European).

In brief, and in light of prior research, we expected grey matter density in bilateral pSMG to correlate with the number of languages spoken on the grounds that this region is sensitive to vocabulary knowledge (Mechelli et al., 2004; Lee et al., 2007; Richardson et al., 2010). On the other hand, we expected grey matter density in the left inferior frontal gyrus to correlate with English lexical efficiency in bilingual speakers, on the grounds that this region is implicated in the paced retrieval of words (Noppeney et al., 2004; Thompson-Schill et al., 1999; Wagner et al., 2001).

## 2 Materials and methods

This study was approved by the joint ethical committee of the Institute of Neurology and the National Hospital for Neurology and Neurosurgery, London, UK.

### 2.1 Participants

Participants were sixty-one, right-handed non-native speakers of English who were resident in the United Kingdom (between 18 and 29 years of age), neurologically normal, and MRI compatible. All participants completed a language background questionnaire (cf. Li, Sepanski, & Zhao, 2006). In line with study aims, they comprised 30 bilinguals (13 male, 17 female) who spoke their native language in addition to English, and 31 multilinguals (10 male, 21 female) who used at least one other language in addition to English and their native language (total range =  3–6 languages). The native language of participants also differed. It was European for 30 and non-European for 31 (see Table 1).

Most (69%) learned English at school (i.e., 37 based on reports from 54 participants). A further 24% (13/54) learned English at school and at home. A final 7% (4/54) acquired English at home or socially. The relative proportions were comparable for bilingual and multilingual speakers and for those whose native language was European or non-European. There was one difference: more participants whose native language was non-European rather than European learned English at both school and home [10:3]. However again there was no marked difference in the number who were bilingual or multilingual speakers [5:8]. Table 1 therefore aggregates over learning history and reports quantitative measures of our participants as a function of our core interest (bilingual/multilingual) and also as a function of their native language (European/non-European). We used univariate analyses of variance to examine how the quantitative measures varied as a function of the number of languages spoken (bilingual/multilingual) and native language (European/non-European), and to check for any interactions between these factors. These analyses showed that, where there were differences as a function of native language background, these did not differ between the bilingual and multilingual groups.

### 2.2 Self-report measures of English language use

Analyses of the participant measures of: age at test, age of English acquisition, years of English use, proportion of current English usage, and self-rated English proficiency [ranging from 1 (low proficiency) to 9 (high proficiency) and averaged over speaking, understanding, reading and writing] showed a good match on all measures between bilingual and mulitlingual speakers. There were no main effects of the number of languages spoken on any of these (all *F* < 1 or *p* > 0.10). There were some differences associated with native language background. Native European language speakers were older than the non-European speakers (*F*(1,57) = 19.25, *p* = 0.001), acquired English later (*F*(1,57) = 19.25, *p* = 0.001), and because they were older, had spoken it for longer (*F*(1,57) = 8.63, *p* = 0.005). Native European language speakers in the sample also used English proportionately more on a daily basis (*F*(1,43) = 4.55, *p* < 0.05). We allude to these differences when we consider the drivers of change in grey matter density. There were no interactive effects of native language background and number of languages spoken on any of these measures (all *F* < 1 or *p* > 0.10).

### 2.3 English behavioral assessments

As a measure of efficiency in English word production, participants completed a letter fluency task in which they generated as many English words beginning with “s” as they could in one minute. As a measure of efficiency in English word recognition, participants completed a computerised lexical decision task. We used material from the PALPA and created non-words by changing one or more letters from real English words (not presented in the task) such that they had plausible spellings in English (i.e., obeyed the orthographic and phonological constraints of English). Following a short practice block, participants responded to a random sequence of 60 word and 60 non-word trials divided into six blocks. Participants pressed one of two response buttons to indicate if the letter string was a word or not. We recorded the accuracy and speed of their responses.

Of these indices, only one differed as a function of the number of languages spoken: for letter fluency, multilinguals scored more highly than bilingual speakers (*F*(1,57) = 4.68, *p* < 0.05; for PALPA accuracy and PALPA reaction time, both Fs < 1). None of these indices varied as a function of native language background (all Fs <1 or *p* > 0.25), nor was there any interaction of native language background with the number of languages spoken for these indices (all Fs < 1 or *p* > 0.15).

In order to compute a measure of efficiency in word recognition, we divided the proportion of accurate response by the mean correct decision time. As a final step, we computed an index of lexical efficiency. Factor analysis of the letter fluency score and the efficiency score for word recognition yielded a single principal component that accounted for 86% of the variance. We used this single composite score as the lexical efficiency regressor along with English age of acquisition in our analysis of brain structure contrasting bilingual and multilingual speakers. We compared the contribution of the different components (lexical decision accuracy, lexical decision response time, and fluency) in post hoc analyses.

### 2.4 MRI imaging and data preprocessing

Structural MRI was acquired using a Siemens Sonata 1.5T scanner (Siemens Medical Systems, Erlangen, Germany). A T1-weighted Modified Driven Equilibrium Fourier Transform (MDEFT) sequence (Deichmann, Schwarzbauer, & Turner, 2004) was used to acquire 176 sagittal partitions with an image matrix of 256 × 224 yielding a final resolution of 1 mm^3^ (TR/TE/TI = 12.24 ms/3.56 ms/530 ms). One T1 anatomical volume was acquired for each participant. Within SPM5 (Wellcome Trust Centre of Imaging Neuroscience; http://www.fil.ion.ucl.ac.uk/spm) running under Matlab 6.5 (MathWorks, Natick, MA), our images were spatially normalized to Montreal Neurological Institute space and segmented into grey and white matter using the unified segmentation algorithm (Ashburner & Friston, 2005) and then spatially smoothed with an isotropic Gaussian kernel of 8 mm at full-width half maximum.

Following segmentation and normalisation, whereby each uniquely shaped brain is rotated and warped to match a common template, the resulting images can be either modulated or unmodulated. Modulated images are corrected for the final volume of the surrounding area as measured by the degree of local compression in the normalization process (i.e., the Jacobian determinates of the deformation). For example, if a brain area was exactly half the size of that in the template, then normalization would require doubling its volume, thereby distributing the intensity of each voxel over twice as much space, and effectively halving each voxel's value. In this case, modulation would correct for this ‘halving’, by effectively multiplying each voxel by two (Mechelli, Friston, Frackowiak, & Price, 2005). In this way, the voxels in modulated images provide an absolute measure of regional volume (Ashburner, 2009). By contrast, unmodulated images are not adjusted for volume, and each voxel provides an estimation of the likelihood the tissue is grey matter relative to white matter and other types of tissue. Crucially, the results of VBM studies often depend on whether modulated or unmodulated images are used. In our previous language studies, for instance, we found our VBM analyses were more sensitive and more consistent across subject groups when we used unmodulated images than when we used modulated images (Grogan, Green, Ali, Crinion, & Price, 2009; Lee et al., 2007; Mechelli et al., 2004; Richardson et al., 2010). This suggests that the effects are in the tissue density rather than volume.

### 2.5 Statistical analyses of structural data

Our results were extracted from one statistical analysis, using a full factorial design implemented in SPM8. This analysis included one factor (group) split between two levels (bilinguals and multilinguals). Two covariates (lexical efficiency and age of English acquisition) were interacted with group, whereas a third (age) was not. This approach allowed us to look for the effect of the covariates and group differences within the same analysis. It also allowed us to regress out the effect of age of acquisition when we compared the two groups, thereby controlling for a potential confound.

There were two kinds of contrast of interest:

1. Contrasts testing for differences between multilingual and bilingual groups
  This contrast involved a direct comparison between brain images for multilingual and bilingual groups. By testing for group differences in the presence of the regressors, this contrast showed where there was an effect of group after lexical efficiency and age of acquisition had been factored out within group, and age had been factored out across group.
2. Contrasts testing for the effect of English lexical efficiency and age of acquisition.
  For the bilingual group only, the multilingual group only, and across both groups, we tested for where grey matter correlated: (a) positively with lexical efficiency only when age of acquisition was factored out; (b) negatively with age of acquisition when lexical efficiency was factored out; and (c) both positively with lexical efficiency and negatively with age of acquisition. Age was factored out in each contrast.

### 2.6 Statistical thresholds

For all the above contrasts (from one SPM analysis), we used a family-wise correction for multiple comparisons (*p* < 0.05 in the size of the peak effect) across the whole brain and also within our regions of interest. The latter were spheres (10 mm radius) centred both on the co-ordinates in left pSMG (*x* = −45, *y* = −59, *z* = +48) and right pSMG (*x* = +56, *y* = −53, *z* = +42) where Mechelli et al. (2004) reported more grey matter density in bilinguals than monolinguals, and in the left middle temporal regions (*x* = +48, *y* = −36, *z* = +6) and (*x* = −50, *y* = −60, *z* = +16) that Richardson et al. (2010) associated with vocabulary knowledge in healthy adult participants.

### 2.7 Post hoc analyses for illustrating the results

For illustrative purposes, we extracted the grey matter signal from each of the identified areas and plotted it against various measures of behavior. This enabled us to illustrate the relationship between grey matter density and the scores for each of the tasks that were used to create the lexical efficiency score. In addition, we used the extracted data in standard SPSS factorial design analyses to test for interactions between multilinguals versus bilinguals and (i) *regions* (e.g., is the effect of multilinguals versus bilinguals greater in the left versus right hemisphere) and (ii) *language background* (e.g., is the effect of multilinguals versus bilinguals greater in those who have a European versus a non-European native language).

## 3 Results

All the findings reported below were identified using unmodulated grey matter images. We found no significant effects in the unmodulated white matter analysis, or when using modulated grey or white matter images, which is consistent with our previous VBM findings (Lee et al., 2007; Richardson, Seghier, Leff, Thomas, & Price, 2011; Richardson et al., 2010).

**Fig. 1:**
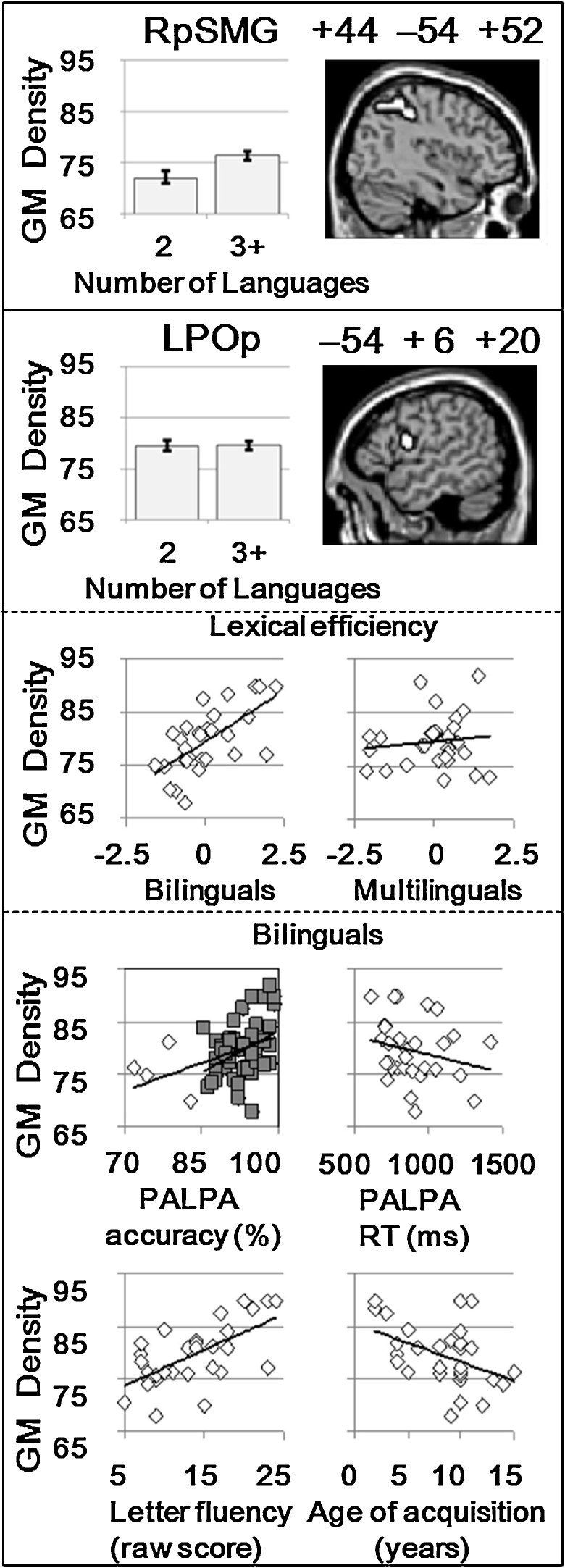
Illustration of the different effects in the right posterior supramarginal gyrus and left pars opercularis. The error bars represent standard errors. Top panel: In right posterior supramarginal gyrus (RpSMG), grey matter density (GM) was higher for multilinguals who spoke 3+ languages (native and non-native) than bilinguals who spoke 2 languages (one native and one non-native). Below: In left pars opercularis (LPOp), GM density did not depend on the number of languages spoken (second panel) but was positively correlated with lexical efficiency in bilinguals but not in multilinguals (third panel). The fourth panel plots components of this index in bilinguals with grey matter density. Grey matter density correlates positively with PALPA accuracy and letter fluency but negatively with PALPA RT and age of acquisition. The plot of PALPA accuracy and grey matter density is presented for (i) all bilinguals (diamond and squares) and (ii) after removing 4 individuals (diamonds) whose accuracy on the PALPA test was more than one standard deviation below the mean.

1. Differences between multilingual and bilingual groups
  In the posterior supramarginal gyrus (pSMG) regions of interest where Mechelli et al. (2004) reported greater grey matter density in bilinguals than monolinguals, we found significantly greater grey matter density in multilinguals (2+ non-native languages) than bilinguals in the right hemisphere (*x* = +44, *y* = −54, *z* = +52; *Z*-score = 3.0; *p* = 0.004 after FWE correction for multiple comparisons in a spherical volume of 10 mm radius centred on the coordinates of Mechelli et al. (2004). This is illustrated on the top, first, panel of Fig. 1. In addition to the significant increase of grey matter in the right pSMG, we also found a trend for grey matter to increase in the left pSMG for multilinguals relative to bilinguals (*x* = −50, *y* = −50, *z* = +46; *Z*-score = 2.5). However, when we followed this up outside of SPM, using a 2 × 2 repeated measures ANOVA with hemisphere (i.e., left pSMG vs right pSMG) and group (bilingual vs multilingual) as factors, there was no significant interaction between hemisphere and group (*p* > 0.05). Consequently, we cannot claim that the increase of grey matter in multilinguals compared to bilinguals was greater in the right pSMG than in the left pSMG. Moreover, we note that pSMG grey matter did not depend on whether the native language was a European or non-European language (*F* < 1).
  There was no significant effect of group (bilingual/multilingual) in the whole brain analysis. Specifically, there were no significant group differences in any part of the left inferior frontal cortex (including the left pars opercularis, LPOp, see Fig. 1, second panel), even when the threshold was lowered to *p* < 0.05 uncorrected.
  Nor did we find any significant effect when we used a small volume correction in the posterior temporal lobe areas where Richardson et al. (2010) reported an effect of vocabulary knowledge in adults. In sum, the only area where grey matter density was significantly higher for multilinguals than bilinguals was the right pSMG where Mechelli et al. (2004) showed more grey matter for bilinguals than monolinguals.
2. Correlations with lexical efficiency and age of acquisition
  In the whole brain analysis, we found a main effect of regressors (higher grey matter with higher lexical efficiency and lower age of acquisition) in LPOp in the left inferior frontal cortex but only in the bilingual group (see Fig. 1, third panel). The peak of this effect was located at MNI co-ordinates (*x* = −54 *y* = +6, *z* = +20) with *Z*-scores of: *Z* = 5.1 in the bilingual group (*p* < 0.05 after FWE correction for multiple comparisons across the whole brain); *Z* = 0.89 in the multilingual group; and *Z* = 3.6 for the interaction of group with regressors.
  We also display how the individual components of that index correlate with grey matter density for bilingual participants where the correlation with lexical efficiency was significant (see Fig. 1, fourth and lower panel). Grey matter density is positively related to the accuracy of lexical decisions in English (*r* = 0.42, *p* = 0.02, for all bilinguals; and *r* = 0.48, *p* = 0.01, excluding those who scored below one standard deviation of the mean, i.e., less than 85%), and to the number of words produced in the letter fluency task (*r* = 0.63, *p* < 0.001). Grey matter density is negatively related to the age of English acquisition (*r* = −0.43, *p* = 0.02) and, though not significantly so, to lexical decision response times (*r* = −0.23, *p* = 0.22). These details quantify the size and direction of effects contributing to the lexical efficiency measure but they should not be over-interpreted as they are not independent of the main SPM analysis where the effect of interest was extracted.
  Neither lexical efficiency nor age of acquisition, in either the bilingual or the multilingual samples, was significantly related to grey matter density in the regions of interest (posterior supramarginal gyri and posterior temporal cortex).
  Our supposition was that the relationship between lexical efficiency and LPOp grey matter density was a (long-term) experience-dependent effect. If so, lexical efficiency should correlate positively with years of English language use which it did (*r* = 0.466, *p* < 0.01). By contrast, lexical efficiency did not correlate with the proportion of current daily use of English (*r* = 0.088). If lexical efficiency is crucial to understanding effects in LPOp, the relationship between lexical efficiency and LPOp grey matter density should remain significant when years of English use (along with age at test and age of English acquisition) are partialled out, which it did (*r* = 0.657, *p* < 0.001).

## 4 Discussion

The present structural brain imaging study has revealed two new results. The first result, and the focus of our discussion, is that the efficiency of non-native lexical processing in English correlates with grey matter density in the left pars opercularis in the left posterior inferior frontal cortex in bilingual speakers. The second result is that multilinguals have higher grey matter density than bilinguals in the posterior supramarginal gyri. These results did not depend on whether the participants’ native language was European or non-European.

The effect of the number of languages on grey matter density in posterior supramarginal gyri (pSMG) is broadly compatible with prior studies though we found significant effects in right pSMG and only a trend in the left pSMG, whereas Mechelli et al. (2004) report the converse. Unlike Mechelli et al. (2004) though we found no correlation between age of English acquisition and grey matter density in pSMG for our bilingual group. We do not think this counts as a failure to replicate because it may be larger sample sizes are needed to detect variation in these regions. Mechelli et al. contrasted 25 early (less than 5 years) and 33 late (between 10 and 15 years) bilinguals, whereas in our sample, using the same criteria, we had 9 early and 15 late bilinguals. We note though that our sample size was sufficient to detect a robust relation between age of English acquisition and grey matter density in left pars opercularis.

Our interpretation of these effects is that grey matter density in the posterior supramarginal gyri indexes the number of words learnt using a formal strategy that links semantics and phonology (Lee et al., 2007; Richardson et al., 2010). Plausibly, multilinguals know more words because they speak more non-native languages than bilinguals. At this point we can only speculate that right pSMG increases the representational resources available for vocabulary acquisition at least when learned using a formal strategy. As in our previous studies (Lee et al., 2007; Richardson et al., 2010), the observed effects were only significant on grey matter density (when the analysis was based on the unmodulated brain images) but not on grey matter volume (when the analysis was based on modulated brain images). This indicates that the effect is arising at the level of tissue type but not at the level of local brain volume.

Our main result highlights an experience-dependent relationship in speakers of more than one language between the efficiency of using English words and grey matter density in the left pars opercularis. Mechelli et al. (2004) explored effects of English language proficiency within bilingual speakers but reported no effect within left pars opercularis conceivably because lexical efficiency but not language proficiency per se emphasises the paced retrieval of lexical knowledge. Our lexical efficiency measure combined the speed and accuracy of word recognition with the number of words produced in a verbal fluency task. There was also a negative relationship between grey matter density and age of acquisition (more grey matter in those who learnt English earlier in life), even though age of acquisition and lexical efficiency were not correlated in our sample. For bilingual speakers, lexical efficiency in English reflects their non-native language processing ability; for multilingual speakers, however, lexical efficiency in English only captures part of their processing ability for non-native languages. A test of the role of this region in multilingual speakers would require lexical efficiency measures in all the non-native languages spoken, but this was not possible to collect in our current sample who spoke so many different languages.

We turn to the functional and structural neuroimaging literature to understand the function of the pars opercularis. Our hypothesis was that lexical efficiency would correlate with grey matter density in frontal regions that are involved in discriminating and selecting amongst alternative lexical candidates (e.g., Noppeney et al., 2004; Thompson-Schill et al., 1999; Wagner et al., 2001). In fact, the location of the inferior frontal area that we have associated with lexical efficiency (*x* = −54, *y* = +6, *z* = +20) is more posterior to the frontal lobe areas associated with the demands on lexical retrieval and selection. Examples of these include: (*x* = −44, *y* = +15, *z* = +22) for selection demands in semantic generation (Thompson-Schill et al., 1999); (*x* = −51, *y* = +18, *z* = +27) for control demands in semantic similarity judgements (Wagner et al., 2001); and (*x* = −42, *y* = +27, *z* = +15) for semantic competition (Noppeney et al., 2004). It is also posterior to a region in Brodmann's areas 45/46 where structure changed with second language learning (Stein et al., 2012).

The closest link we could find between the pars opercularis and lexical interference was a study by Heim, Eickhoff, Friederici, and Amunts (2009) who reported increased activation at (*x* = −50, *y* = +8, *z* = +19) during picture naming, when the pictures to be named were blocked according to similarity in semantics or gender. In this context, increased activation in the pars opercularis could either reflect increased semantic interference or increased reliance on other processes such as phonology.

The association of the left pars opercularis with phonology has been well established by previous studies, as functional imaging and repetitive transcranial magnetic stimulation (rTMS) studies have shown that the left pars opercularis is activated by, and necessary for, phonemic processing and subvocal rehearsal in verbal working memory (Burton, Small, & Blumstein, 2000; Gough, Nobre, & Devlin, 2005; Hartwigsen et al., 2010; Nixon, Lazarova, Hodinott-Hill, Gough, & Passingham, 2004; Paulesu, Frith, & Frackowiak, 1993; Zatorre, Meyer, Gjedde, & Evans, 1996). For example, Myers, Blumstein, Walsh, & Eliassen (2009) recently reported activation at (*x* = −41, *y* = +8, *z* = +27) in response to pre-lexical stimuli when there is an unexpected change in phonetic category. Of most relevance to our findings is the observation that the surface area of the left pars opercularis correlates with phonetic transcription ability in the native language of phoneticians (Golestani, Price, & Scott, 2011), and structural differences in this region (together with structural differences in auditory and parietal cortices) help predict individual differences in the ease of perceiving and producing foreign speech sounds (Golestani, Molko, Dehaene, LeBihan, & Pallier, 2007; Golestani & Pallier, 2007; Golestani, Paus, & Zatorre, 2002).

Why might the pars opercularis be required for efficient processing of non-native words? Previous studies have shown that left pars opercularis is activated during covert as well as overt speech production tasks (Callan et al., 2006; Carreiras, Mechelli, Estévez, & Price, 2007; Indefrey & Levelt, 2004) and for retrieving the names of objects and words relative to an articulation task that does not involve lexical retrieval (Parker Jones et al., 2012). Acquiring and using a second language increases the demand on the resources required for lexical retrieval as behavioural, ERP, and neuroimaging data show that there is joint activation of words in each language (see, for example, Bialystok, Craik, Green, & Gollan, 2009; Kroll, Bobb & Wodniecka, 2006; van Heuven & Dijkstra, 2010 for reviews). Indeed, in Parker Jones et al. (2012), we found that activation in POp was higher during lexical retrieval in bilinguals than monolinguals. We infer that increased demand drives plastic changes in grey matter density. Such changes are not linearly related to the number of languages spoken as there was no group effect in this region. However, the neural mechanisms these changes index, and that permit increased efficiency in lexical processing, are unknown.

In summary, we have reported two new findings that extend our understanding of how brain structure is related to language ability. Grey matter density in the left pars opercularis correlates with lexical efficiency in English for bilingual speakers but not with the number of languages known whereas grey matter density in the posterior supramarginal gyri reflects the sheer number of words known.

## Figures and Tables

**Table 1:** Participant details [including means and standard deviations in brackets] with the results of lexical tasks in English.

Participant group^a^	Bilingual			Multilingual			EL: non-EL
	European	Non-European	Sum/mean(SD)	European	Non-European	Sum/mean(SD)	Sum/mean(SD)
**Native language**	16	14	30	13	18	31	29:32
Male:female	6:10	7:7	13:17	6:7	4:14	10:21	12:17/11:21
Age at test (years)	30.9 (7.1)	21.9 (4.1)	26.7 (7.4)	30.6 (8.1)	24.2 (7.3)	26.9 (8.2)	30.8(7.4): 23.2 (6.1)
**English**							
Self-rated ability (scale 1–9)	8.3 (0.9)	7.8 (1.8)	8.1 (1.3)	8.3 (0.8)	8.4 (0.5)	8.4 (0.7)	8.3 (0.9): 8.1 (1.3)
Age of English acquisition (years)	9.1 (2.6)	7.4 (4.2)	8.3 (3.5)	8.3 (4.2)	4.9 (3.7)	6.4 (4.2)	8.7 (3.4): 6.0 (4.1)
Years of use	21.9 (7.2)	14.6 (5.2)	18.5 7.3)	22.3 (8.4)	19.2 (6.4)	20.5 (7.3)	22.1 (7.6): 17.2 (6.3)
**Current use of languages: % of day**^b^							
Native language	39.6 (16.2)	62.0 (23.5)	48.5 (22.0)	35.8 (15.9)	35.2 (19.9)	35.5 (17.4)	37.9 (15.8): 48.6 (25.3)
English	58.3 (17.0)	38.0 (23.5)	50.2 (21.9)	53.6 (14.1)	50.0 (21.5)	51.9 (17.5)	56.2 (15.7): 44.0 (22.8)
Other	–	–	–	7.3 (16.8)	13.1 (12.7)	10.0 (15.0)	
**Lexical tasks in English**							
Letter fluency	13.4 (5.3)	13.5 (5.7)	13.5 (5.4)	15.7 (4.1)	17.2 (5.8)	16.6 (5.2)	14.4 (4.9): 15.6 (5.9)
**Lexical decision (PALPA)**							
Accuracy	92.9 (5.8)	90.2 (7.9)	91.7 (6.9)	91.3 (4.5)	89.8 (8.7)	90.4 (7.2)	92.2 (5.2): 90.0 (8.3)
RT (ms)	849 (136)	945 (242)	894 (196)	922 (215)	872 (200)	893 (204)	882 (175): 904 (219)

a Bilinguals – speak one non-native language. Multilinguals – speak more than one non-native language. Participants also differed in their native languages. European languages (EL) include Dutch, French, German, Greek, Portuguese and Spanish; non-European languages (non-EL) include Cantonese, Gujerati, Hakka, Hebrew, Hindi, Hokkien and Mandarin.

b The percentage estimates of each language used do not always sum to 100 because individuals did not invariably ensure that they did so.
